# Evaluation of actual and estimated hydraulic conductivity of sands with different gradation and shape

**DOI:** 10.1186/s40064-016-2472-2

**Published:** 2016-06-21

**Authors:** Ali Firat Cabalar, Nurullah Akbulut

**Affiliations:** Department of Civil Engineering, University of Gaziantep, 27310 Gaziantep, Turkey; Department of Civil Engineering, Hasan Kalyoncu University, 27100, Gaziantep Turkey

**Keywords:** Hydraulic conductivity, Sand, Gradation, Shape

## Abstract

Hydraulic conductivities of sands with different gradation and grain shape were estimated experimentally at a relative density (D_r_) of about 40 % and a 22 ± 2 °C of constant temperature. Narli Sand (NS) with 0.67 of sphericity (S) and 0.72 of roundness (R), and Crushed Stone Sand (CSS) with 0.55 of S and 0.15 of R values were artificially graded into sixteen different grain-size fractions (4.75–2, 2–1.18, 1.18–0.6, 0.6–0.425, 0.425–0.3, 0.3–0.075, 4.75–0.075, 2–0.075, 1.18–0.075, 0.6–0.075, 0.425–0.075, 4.75–0.6, 2–0.6, 4.75-0.425, 2–0.425, 1.18–0.425 mm). Hydraulic conductivities of the NS estimated by use of constant head test ranged from 1.61 to 0.01 cm/s, whilst those of the CSS estimated by the same test ranged from 2.45 to 0.012 cm/s. It was observed that the hydraulic conductivity values of the NS are lower than those of the CSS samples, which is likely to be the result of differences in shape, particularly in R values. The results clearly demonstrated that the hydraulic conductivity can be significantly influenced by grading characteristics (d_10_, d_20_, d_30_, d_50_, d_60_, c_u_, c_c_, n, I_o_). Furthermore, comparisons between results obtained in the present study and hydraulic conductivity estimated with other formulas available in the literature were made. The comparisons indicated that the best estimation of hydraulic conductivity changes based on the gradation and shape properties of the sands tested.

## Background

Hydraulic conductivity, which represents the ability of a porous media to transmit water through its voids, is one of the most significant key parameters of geomaterials for many natural phenomena including the management of water resources, drinking water supply, safety of waste repositories, basin-scale hydrogeologic circulation, stability analyses, and many other problems on subsurface hydrology and geotechnical engineering (Terzaghi and Peck 1964; Moore et al. 1982; Wintsch et al. 1995; Person et al. 1996; Boadu 2000; Chapuis 2012). There have been attempts to estimate hydraulic conductivity based on grain size distribution (Mualem 1976; Freeze and Cherry 1979; Uma et al. 1989; Salarashayeri and Siosemarde 2012). Empirical (Hazen 1911; Krumbein and Monk 1942; Alyamani and Sen 1993) and predictive methods (Kozeny 1927; Carman 1937; Boadu 2000; Goktepe and Sezer 2010) of estimating the hydraulic conductivity using quantitative relations have been developed in the literature. A commonly accepted equation was proposed by Hazen (1911) and given *k* = *cd*_10_^2^ for predicting the hydraulic conductivity of saturated sands. Where *k* is hydraulic conductivity, *c* is constant, and *d*_10_ is effective diameter at which 10 % of the grains are finer. Krumbein and Monk (1942) gave an expression for the hydraulic conductivity of unconsolidated sands by an empirical equation of the form *k* = (760*d*_*w*_^2^)exp(−1.3*σ*_*ψ*_), where *d*_*w*_ is geometric mean diameter by weight in millimetres, *σ*_*ψ*_ is standard deviation of the *ψ* distribution function. Masch and Denny (1966) proposed the use of *d*_50_ median grain size as the representative size to correlate hydraulic conductivity with grain size. Kozeny (1927) and Carman (1937), which is widely accepted derivation for hydraulic conductivity, developed a semi-empirical formula for predicting the permeability of porous media. Koltermann and Gorelick (1995) stated that the use of geometric mean overpredicts hydraulic conductivity by several orders of magnitude for soils with significant fines content, whilst the harmonic mean grain size under predicts *k* by several orders of magnitude for soils with less fines content. Shepherd (1989) performed a series of statistical power regression analyses on 19 sets of published data on hydraulic conductivity of unconsolidated sediments versus grain size. Alyamani and Sen (1993) proposed an equation based on analysis of 32 samples incorporating the initial slope and the intercept of the grain-size distribution curve. Sperry and Peirce (1995) developed a model for delineating the significance of particle size/shape, and porosity in explaining the variability of hydraulic conductivity of a granular porous medium. Ishaku et al. (2011) have employed several empirical formulae to specify the hydraulic conductivity of aquifer materials in the field. Although many different techniques have been proposed to determine hydraulic conductivity value, including field methods, applications of these empirical formulae to the same porous medium material can yield different values of hydraulic conductivity because of the difficulty of including all possible variables in porous media (Vukovic and Soro 1992).

**Fig. 1:**
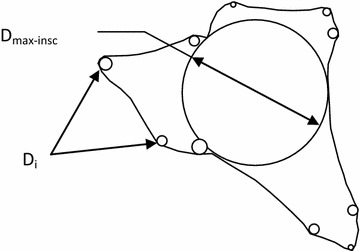
Graphical representation of roundness, R (redrawn from Muszynski and Stanley, 2012)

**Fig. 2:**
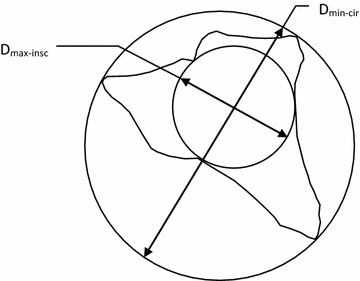
Graphical representation of sphericity, S (redrawn from Muszynski and Stanley, 2012)

**Fig. 3:**
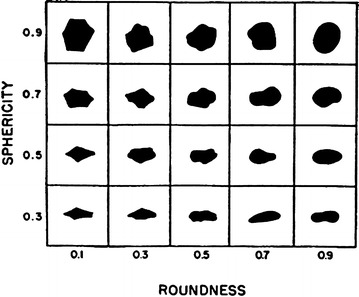
Comparison chart (Santamarina and Cho 2004)

It has been long understood that grain shape characteristics have a significant effect on certain engineering properties of soils (Terzaghi 1925; Gilboy 1928; Lees 1964; Olson and Mesri 1970; Abbireddy et al. 2009; Clayton et al. 2009). Terzaghi is one of the first engineers to perform a research to understand the influences of shape characteristics by employing flat-grained constituents (Terzaghi 1925). The observations, conducted by Gilboy (1928), that any system of analysis neglecting the effect of grain shape would be incomplete. Numerous researches have been conducted due to the significance of grains’ shape and its role in the behaviour of soils for both practicing engineers and researchers. Holubec and D’Appolonia (1973) indicated that the results of dynamic penetration tests in sands depend on grains’ shape characteristics. Cornfort (1973), and Holtz and Kovacks (1981) pointed out how grain shape affects the internal friction angle (φ). Cedergen (1989) stated that grain shape affects the permeability. Grain shape also plays an important role in liquefaction potential (Kramer 1996). Wadell (1932), Krumbein (1941), Powers (1953), Holubec and D’Appolonia (1973), Youd (1973), and Cho et al. (2006) have introduced detailed explanations of grain shape. Two independent properties are basically used to describe the shape of a soil grain: (1) Roundness, a measure of the extent to which the edges and corners of a grain has been rounded (2) Sphericity (form), a measure of the extent to which a grain approaches a sphere in shape. Wadell (1932) proposed a simplified sphericity (S) parameter (D_max-insc_/D_min-circ_), where D_max-min_ is the diameter of a maximum inscribed circle and D_min-circ_ is the diameter of a minimum sphere circumscribing a gravel particle. Wadell (1932) defined roundness (R) as D_i-ave_/D_max-insc_, where D_i-ave_ is the average diameter of the inscribed circle for each corner of the particle. Figures 1, 2 and 3 describe R, S and a chart for comparison between them to identify grain shape (Krumbein 1941; Powers 1953).

Although many field and laboratory determinations of hydraulic conductivity have been performed by engineers, geologist, hydrogeologist, and soil scientists, the fundamental relationships between the gradation and shape properties of grains and flow through them remain poorly understood and inadequately quantified. Actually, these approaches cannot yield consistent results with respect to actual hydraulic conductivity values. Therefore, this study aims to evaluate a new conceptual approach for quantifying the inherent coupling between gradation/shape of sand grains changes and hydraulic conductivity by exploiting constant head permeability tests on sixteen different grain-size fractions (4.75–2, 2–1.18, 1.18–0.6, 0.6–0.425, 0.425–0.3, 0.3–0.075, 4.75–0.075, 2–0.075, 1.18–0.075, 0.6–0.075, 0.425–0.075, 4.75–0.6, 2–0.6, 4.75–0.425, 2–0.425, 1.18–0.425 mm) of sands having two distinct shapes (rounded and angular). Furthermore, comparisons between results obtained in the present study and hydraulic conductivity estimated with other formulas available in the literature were made.

## Experimental study

**Fig. 4:**
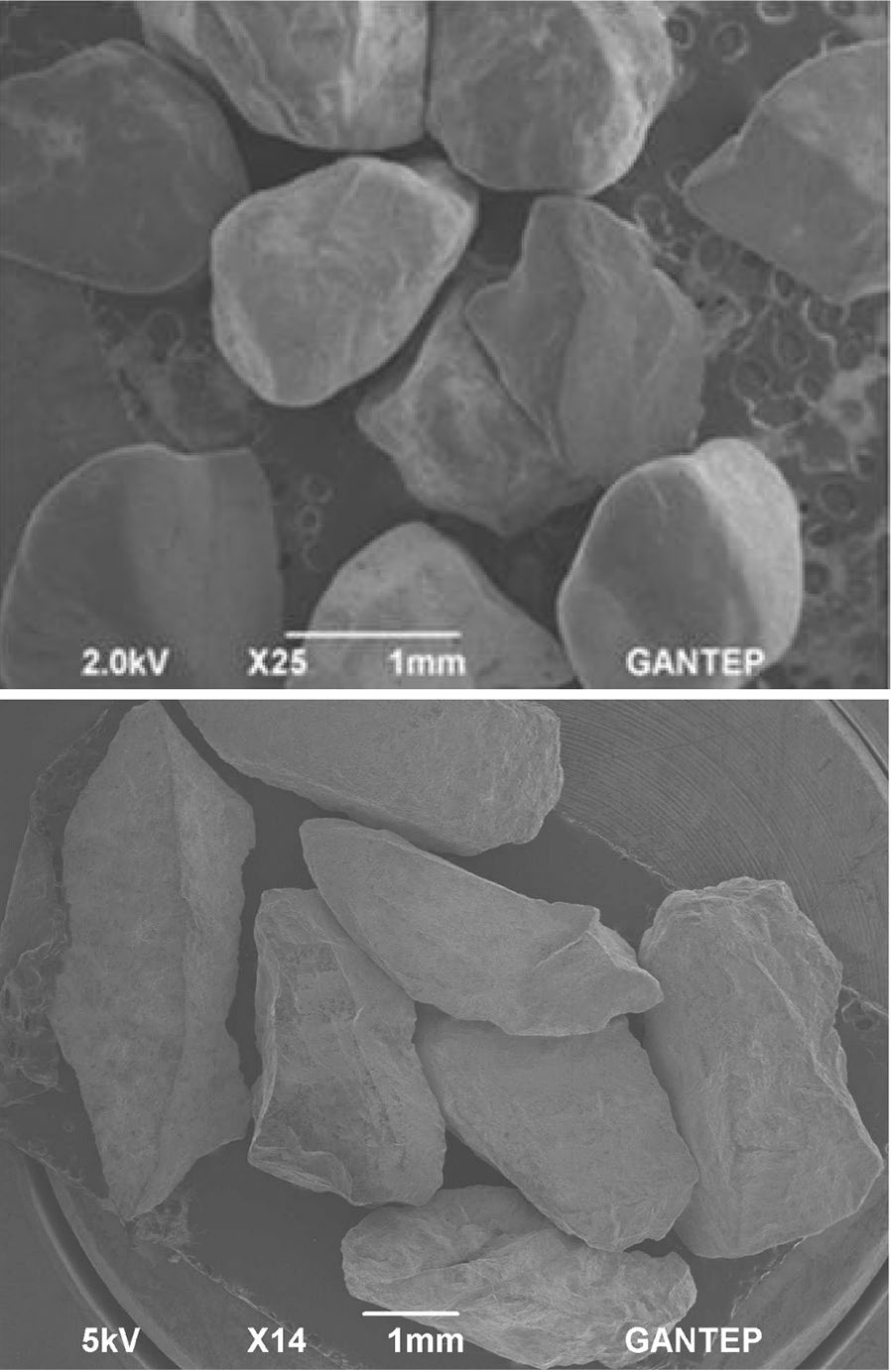
SEM pictures of the (*top*) CSS and (*bottom*) NS used during the experimental study

**Fig. 5:**
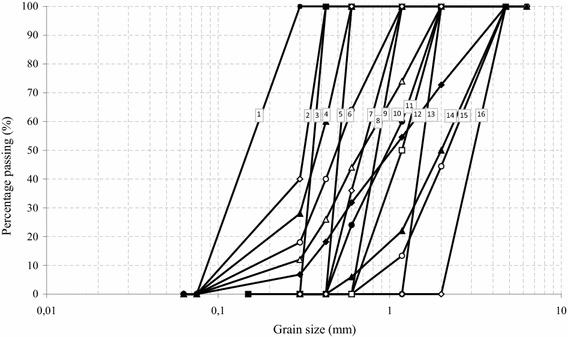
Grain size distributions for the sands used during the experimental study

The materials used in the tests described in this study were Narli Sand (NS) and Crushed Stone Sand (CSS) having the distinct shapes and sizes falling between 4.75 and 2 mm, 2 and 1.18 mm, 1.18 and 0.6 mm, 0.6 and 0.425 mm, 0.425 and 0.3 mm, 0.3 and 0.075 mm, 4.75 and 0.075 mm, 2 and 0.075 mm, 1.18 and 0.075 mm, 0.6 and 0.075 mm, 0.425 and 0.075 mm, 4.75 and 0.6 mm, 2 and 0.6 mm, 4.75 and 0.425 mm, 2 and 0.425 mm, 1.18 and 0.425 mm. Narli Sand (NS) was quarried in and around Narli, Kahramanmaras in southern-central of Turkey. A commercially available Crushed Stone Sand (CSS) was supplied from the same region of Turkey, which is widely consumed in earthworks in the region. The specific gravity of the grains were found to be 2.65 for Narli Sand, and 2.68 for Crushed Stone Sand. Scanning Electron Micrograph (SEM) pictures show the physical differences/similarities among the sands used during this investigation (Fig. 4). As can be seen from the Fig. 4, Narli Sand grains have rounded, whereas the Crushed Stone Sand grains have angular shape. Figure 5 indicates the grain size distribution of the sands used during the experimental study. Roundness (R) and sphericity (S) estimations based on the study by Muszynski and Stanley (2012) were found to be 0.43, 0.67, and 0.16, 0.55 for the NS and CSS grains, respectively. The sands were tested in a constant head permeability testing apparatus at a relative density (R_d_) of about 40 % and constant room temperature (20 ± 2 °C). The specimens, which were placed in a perspex cylindrical cell of about 50 cm^2^ cross-sectional area (*A*), rest on a wire mesh at bottom of the cell. The volume of the water (*q*) flowing during a certain time (*t*) is measured, when a steady vertical water flow, under a constant head, is maintained through the soil specimen. Then, *k* values of the specimens tested were calculated using Darcy’s law (*k* = *ql*/*Ah*). Tables 1 and 2 present some physical characteristics of the NS and CSS samples, respectively. As can be seen from these tables the hydraulic conductivity is affected by grading characteristics d_10_, d_20_, d_30_, d_50_, d_60_, c_u_, c_c_, n, and I_o_.

**Table 1:** Some physical characteristics of the NS samples

Gradation (mm)	d_10_ (mm)	d_20_ (mm)	d_30_ (mm)	d_50_ (mm)	d_60_ (mm)	c_u_	c_c_	n	e_max_	e_min_	e_test_	d (g/cm^3^)	I_0_ (mm)	k (cm/s)
4.75–2	2.20	2.40	2.60	3.10	3.30	1.50	0.93	0.46	0.95	0.70	0.86	1.44	2.00	1.61
2–1.18	1.25	1.30	1.38	1.63	1.70	1.35	0.89	0.45	0.92	0.66	0.82	1.47	1.18	0.35
1.18–0.6	0.64	0.69	0.74	0.84	0.90	1.41	0.93	0.44	0.85	0.62	0.77	1.51	0.60	0.13
0.6–0.425	0.43	0.45	0.47	0.50	0.51	1.19	1.00	0.42	0.79	0.60	0.72	1.56	0.43	0.05
0.425–0.3	0.32	0.33	0.34	0.35	0.37	1.16	0.98	0.37	0.61	0.52	0.60	1.70	0.30	0.03
4.75–0.075	0.33	0.44	0.59	1.00	1.45	4.39	0.73	0.47	0.97	0.70	0.87	1.43	0.25	0.03
2–0.075	0.24	0.36	0.46	0.69	0.87	3.63	1.01	0.45	0.92	0.63	0.82	1.47	0.19	0.02
1.18–0.075	0.17	0.31	0.38	0.50	0.59	3.47	1.44	0.44	0.89	0.61	0.79	1.50	0.14	0.02
0.6–0.075	0.13	0.20	0.31	0.39	0.43	3.27	1.74	0.41	0.80	0.52	0.70	1.57	0.09	0.02
0.425–0.075	0.11	0.16	0.22	0.32	0.34	3.09	1.29	0.39	0.74	0.44	0.63	1.64	0.08	0.01
0.3–0.075	0.09	0.10	0.12	0.16	0.18	2.07	0.92	0.36	0.63	0.42	0.56	1.72	0.08	0.01
4.75–0.6	1.00	1.33	1.60	2.20	2.60	2.60	0.98	0.43	0.83	0.59	0.74	1.54	0.82	0.26
2–0.6	0.69	0.79	0.90	1.18	1.33	1.93	0.88	0.40	0.76	0.52	0.67	1.60	0.60	0.12
4.75–0.425	0.70	1.10	1.40	2.00	2.30	3.28	1.22	0.34	0.57	0.42	0.52	1.77	0.55	0.14
2–0.425	0.49	0.58	0.68	0.99	1.18	2.41	0.80	0.41	0.78	0.55	0.70	1.58	0.41	0.06
1.18–0.425	0.46	0.51	0.58	0.70	0.79	1.71	0.93	0.36	0.61	0.46	0.55	1.72	0.43	0.04

**Table 2:** Some physical characteristics of the CSS samples

Gradation (mm)	d_10_ (mm)	d_20_ (mm)	d_30_ (mm)	d_50_ (mm)	d_60_ (mm)	c_u_	c_c_	n	e_max_	e_min_	e_test_	d (g/cm^3^)	I_0_ (mm)	k (cm/s)
4.75–2	2.20	2.40	2.60	3.10	3.30	1.50	0.93	0.51	1.12	0.83	1.02	1.32	2.00	2.45
2–1.18	1.25	1.30	1.38	1.63	1.70	1.35	0.89	0.50	1.08	0.82	0.99	1.34	1.18	0.47
1.18–0.6	0.64	0.69	0.74	0.84	0.90	1.41	0.93	0.49	1.04	0.80	0.96	1.36	0.60	0.19
0.6–0.425	0.43	0.45	0.47	0.50	0.51	1.19	1.00	0.48	0.88	0.75	0.83	0.45	0.43	0.11
0.425–0.3	0.32	0.33	0.34	0.35	0.37	1.16	0.98	0.45	1.08	0.80	0.98	1.34	0.30	0.07
4.75–0.075	0.33	0.44	0.59	1.00	1.45	4.39	0.73	0.48	1.02	0.69	0.91	1.40	0.25	0.05
2–0.075	0.24	0.36	0.46	0.69	0.87	3.63	1.01	0.48	1.03	0.69	0.91	1.40	0.19	0.03
1.18–0.075	0.17	0.31	0.38	0.50	0.59	3.47	1.44	0.46	0.97	0.64	0.86	1.43	0.14	0.03
0.6–0.075	0.13	0.20	0.31	0.39	0.43	3.27	1.74	0.44	0.91	0.56	0.79	1.49	0.09	0.02
0.425–0.075	0.11	0.16	0.22	0.32	0.34	3.09	1.29	0.43	0.86	0.52	0.74	1.53	0.08	0.02
0.3–0.075	0.09	0.10	0.12	0.16	0.18	2.07	0.92	0.40	0.78	0.47	0.67	1.59	0.08	0.01
4.75–0.6	1.00	1.33	1.60	2.20	2.60	2.60	0.98	0.46	0.97	0.66	0.86	1.44	0.82	0.29
2–0.6	0.69	0.79	0.90	1.18	1.33	1.93	0.88	0.44	0.91	0.60	0.80	1.48	0.60	0.22
4.75–0.425	0.70	1.10	1.40	2.00	2.30	3.28	1.22	0.41	0.78	0.57	0.71	1.56	0.55	0.19
2–0.425	0.49	0.58	0.68	0.99	1.18	2.41	0.80	0.47	0.99	0.70	0.89	1.41	0.41	0.09
1.18–0.425	0.46	0.51	0.58	0.70	0.79	1.71	0.93	0.42	0.80	0.57	0.71	1.55	0.43	0.09

## Results and discussion

Table 3 gives a summary of the specimens used in the tests reported here. The initial relative densities of all specimens were around 40 %. The specimens were loose to medium dense. Sixteen different sizes of artificially graded NS and CSS sands, which have exactly the same gradation characteristics (*d*_10_, *d*_20_, *d*_30_, *d*_50_, *d*_60_, *c*_*u*_, *c*_*c*_, *I*_*o*_) (Fig. 5) within the specified ranges, have been classified as ‘poorly graded’ (*SP*) based on the Unified Soil Classification System (USCS9. Based on the roundness criteria and values proposed by Powers (1953), and Youd (1973), the specimens used during the experimental investigation were found to be very angular and rounded for CSS and NS grains, respectively.

**Table 3:** Summary of specimen data

Gradation (mm)	Hydraulic conductivity (k, cm/s)																	
	Hazen		K–C		Terzaghi		Chapuis		Slitcher		NAVFAC		USBR		A–S		Breyer	
	NS	CSS	NS	CSS	NS	CSS	NS	CSS	NS	CSS	NS	CSS	NS	CSS	NS	CSS	NS	CSS
4.75–2	5.95	8.39	4.93	12.36	2.38	4.85	6.31	6.33	1.78	3.55	8.48	13.24	4.46	4.46	6.16	6.16	7.21	7.21
2–1.18	2.39	2.95	2.79	5.19	1.20	1.89	1.87	1.86	0.88	1.37	3.16	5.37	1.09	1.09	2.13	2.13	2.37	2.37
1.18–0.6	0.67	0.79	0.86	1.49	0.35	0.53	0.48	0.49	0.26	0.38	0.87	1.69	0.25	0.25	0.55	0.55	0.62	0.62
0.6–0.425	0.32	0.37	0.46	0.74	0.18	0.25	0.21	0.22	0.13	0.18	0.47	0.93	0.09	0.09	0.27	0.27	0.29	0.29
0.425–0.3	0.18	0.21	0.29	0.44	0.11	0.15	0.12	0.12	0.08	0.11	0.31	0.62	0.05	0.05	0.14	0.14	0.16	0.16
4.75–0.075	0.13	0.16	0.10	0.16	0.05	0.07	0.12	0.11	0.04	0.05	0.08	0.14	0.09	0.09	0.11	0.11	0.13	0.13
2–0.075	0.08	0.09	0.07	0.11	0.03	0.05	0.06	0.06	0.02	0.03	0.06	0.10	0.06	0.06	0.06	0.06	0.07	0.07
1.18–0.075	0.04	0.05	0.05	0.07	0.02	0.03	0.03	0.03	0.02	0.02	0.04	0.06	0.04	0.04	0.03	0.03	0.04	0.04
0.6–0.075	0.03	0.03	0.04	0.05	0.02	0.02	0.02	0.02	0.01	0.01	0.03	0.05	0.02	0.02	0.02	0.02	0.02	0.02
0.425–0.075	0.02	0.03	0.03	0.04	0.01	0.01	0.01	0.01	0.01	0.01	0.03	0.05	0.01	0.01	0.01	0.01	0.02	0.02
0.3–0.075	0.01	0.02	0.02	0.03	0.01	0.01	0.01	0.01	0.01	0.01	0.02	0.04	0.01	0.01	0.01	0.01	0.01	0.01
4.75–0.6	1.16	1.51	0.89	1.73	0.44	0.75	1.18	1.15	0.33	0.55	1.17	1.89	1.15	1.15	1.09	1.09	1.35	1.35
2–0.6	0.71	0.87	0.78	1.44	0.34	0.54	0.54	0.54	0.25	0.39	0.79	1.54	0.35	0.35	0.56	0.56	0.70	0.70
4.75–0.425	0.52	0.74	0.36	0.83	0.18	0.36	0.57	0.57	0.14	0.27	0.43	0.85	0.74	0.74	0.51	0.51	0.63	0.63
2–0.425	0.34	0.40	0.36	0.56	0.16	0.22	0.27	0.26	0.12	0.16	0.34	0.56	0.17	0.17	0.27	0.27	0.33	0.33
1.18–0.425	0.33	0.38	0.40	0.59	0.17	0.23	0.24	0.24	0.13	0.16	0.39	0.62	0.13	0.13	0.28	0.28	0.31	0.31

**Fig. 6:**
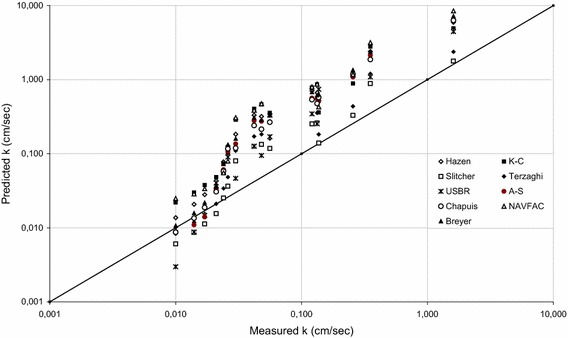
Comparison of measured hydraulic conductivity (k) with predictions from various models for NS samples (*straight line* represents line of perfect equality)

**Fig. 7:**
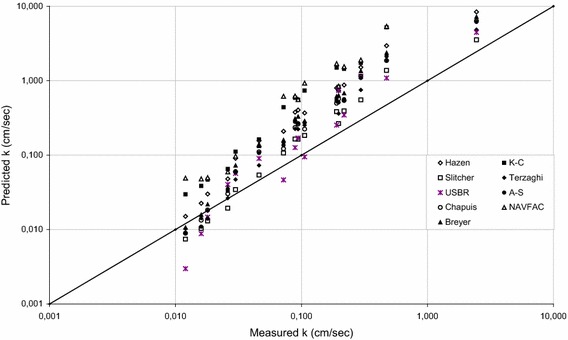
Comparison of measured hydraulic conductivity (k) with predictions from various models for CSS samples (*straight line* represents line of perfect equality)

Table 4 shows the empirical equations and their limitations for hydraulic conductivity estimates which were used to obtain the results given in Table 3. Equations developed by Hazen (1892), Kozeny-Carman (1956), Terzaghi (Odong 2007), Chapuis (2004), Slichter (1898), USBR (Vukovic and Soro 1992), NAVFAC (1974), Alyamani and Sen (1993), and Breyer (Kresic 1998) were employed in this study. Hazen (1892) proposed his formula in order to estimate the hydraulic conductivity of uniformly graded loose sand with effective grain size (*d*_10_) between 0.10 and 3.0 mm, and *c*_*u*_ less than 5. As can be seen from the Table 3 that hydraulic conductivity values ranged from 5.95 to 0.01 cm/s for the NS samples falling specified gradations, whilst those ranged from 8.39 to 0.02 cm/s for the CSS samples falling the same gradations. Although, presence of porosity (*n*) in the formula seems an advantage of the formula, this approach does not give an accurate estimates for the sands due to the limits of *c*_*u*_ indicated in Table 4. The authors consider that influence of the parameter *c*_*u*_ was neglected in his study, and thereby the grain size distribution results could yield the same *c*_*u*_ for various sands. Kozeny–Carman (K–C) formula, which is not applicable for neither clayey soils nor soils with effective size more than 3 mm, is one of the commonly employed approaches developed for hydraulic conductivity estimates (Carrier 2003). Actually, the Kozeny (1927) and Carman (1937) equations have been modified by certain researchers (Collins 1961; Bear 1972; de Marsily 1986), whom included the influence of both particle diameter and porosity on hydraulic conductivity. Koltermann and Gorelick (1995) compared five different approaches and found that the original Kozeny–Carman equation (Carman 1937; Bear 1972) lies approximately in the center of the possible relations. Koltermann and Gorelick (1995) used the geometric and harmonic means to calculate representative particle diameters for the high and low fraction of the coarse component, respectively. However, this approach produces a discontinuity when the fraction of the coarse component is at the intermediate level. Therefore, the authors employed the original Kozeny–Carman equation, then the Table 3 released that hydraulic conductivity values ranged from 4.93 to 0.02 cm/s for the NS samples, while those ranged from 12.36 to 0.03 cm/s for the CSS samples falling the same gradations. Estimated hydraulic conductivity values (*k*) by employing Terzaghi’s approach varied from 2.38 to 0.01 cm/s for the NS samples, whilst the *k* values varied from 4.85 to 0.01 cm/s for CSS samples. Cheng and Chen (2007) pointed out that Terzaghi’s formula is most applicable for large-grain sand. However, comparing the experimental results and the *k* values obtained via Terzaghi’s approach revealed that Terzaghi’s equation, which has no limitations reported (Table 4), gives more accurate results than the other equations employed for both NS and CSS samples between 1.18 and 0.075 mm, and 0.6 and 0.075 mm. Surprisingly, it gives much less accurate results for larger grains of both NS and CSS samples, including the size of 4.75–2, 2–1.18, and 4.75–0.425 mm. Therefore, the authors interpreted that grain size would not be the only parameter to make an accurate hydraulic conductivity estimate. Estimated *k* values via Chapuis formula gives the best correlation with measured *k* values for the NS samples between 0.425 and 0.075 mm. Generally speaking, estimated *k* values using Chapuis’s approach ranged from 6.31 to 0.01 cm/s for the NS samples, whilst those ranged from 6.33 to 0.01 cm/s for the CSS samples falling the same gradations. In the light of the Goktepe and Sezer (2010), which indicated that Chapuis method best estimates the hydraulic conductivity of fine sands, the predictions were found to be acceptable for the NS samples but not for the CSS samples. The authors considered that such difference could be because of shape properties of the sand grains. Although Goktepe and Sezer (2010) indicated that the Chapuis and Slitcher approaches are in harmony with the results, the present study shows remarkable differences between these two approaches. Considering the differences in relative density values employed in these studies, the authors’ interpretation is that such differences in the approaches could be the reason of high successes of the empirical equations. For example, the present study shows that Slitcher formula is the best fitted to the hydraulic conductivity of NS samples between 4.75 and 2 mm, 2 and 1.18 mm, 4.75 and 0.075 mm, 2 and 0.075 mm, 4.75 and 0.6 mm, 2 and 0.6 mm, 4.75 and 0.425 mm, 2 and 0.425 mm, 1.18 and 0.425 mm, and the hydraulic conductivity of CSS samples between 4.75 and 2 mm, 4.75 and 0.075 mm, 2 and 0.075 mm, 4.75 and 0.6 mm, 4.75 and 0.425 mm, 2 and 0.425 mm. However, Chapuis approach does not give similar results. The Naval Facilities Engineering Command (NAVFAC) suggested a chart to estimate the hydraulic conductivity of clean sand and gravel based on the *e* and *d*_10_. Predicted *k* values using NAVFAC varied from 8.48 to 0.01 cm/s for the NS samples, and 13.24 to 0.04 cm/s for the CSS samples. The approach proposed by the United States Bureau of Reclamation (USBR 1990) estimates *k* values using the effective grain size (*d*_20_), and it does not depend on the porosity (Table 4). Cheng and Chen (2007) stated that this approach is most suitable for medium-grain sand with *c*_*u*_ less than 5. Estimated *k* values using the USBR formula were found to be same for NS samples and CSS samples, which ranged from 4.46 to 0.01 cm/s, as they have the same gradations. It was observed that the USBR approach gave its best results for relatively large grain samples including those between 2 and 1.18 mm, 1.18 and 0.6 mm, 0.6 and 0.425 mm, and 1.18 and 0.425 mm. Alyamani and Sen (A–S), which is one of the widely known approaches to estimate the hydraulic conductivity, employs the grain size properties *d*_10_, *d*_50_ and *I*_*o*_. Alyamani and Sen (1993) proposed their equation based on different samples that incorporates the initial slope and the intercept of the grading curve. Estimated *k* values using the Alyamani and Sen approach ranged from 6.16 to 0.01 cm/sec for both type of sands. As can be seen from Table 3 that the A–S approach results in same estimates for both NS and CSS samples, as they have same grading curves. Similarly, Breyer method gave the same *k* values for both NS and CSS samples due to the same *d*_10_ value employed in this equation. The predicted *k* values ranged from 7.21 to 0.01 cm/s. Plots presented in Figs. 6 and 7 indicate comparisons of measured hydraulic conductivity (*k*) with predictions from various models for NS samples, and CSS samples, respectively.

**Table 4:** Empirical equations and their limitations for permeability estimates

Researcher/organization	Equation	Limitations
Hazen	$k = 6 \times 10^{ - 4} \times \frac{g}{v} \times [1 + 10(n - 0.26)] \times (d_{10} )^{2}$	C_u_ < 5 0.1 < d_10_ < 3.0
Kozeny-Carman	$k = 8.3 \times 10^{ - 3} \times \frac{g}{v} \times \left[ {\frac{{n^{3} }}{{(1 - n)^{2} }}} \right] \times (d_{10} )^{2}$	0.5 < d_10_ < 4.0
Terzaghi	$k = 0.0084 \times \frac{g}{v} \times \left[ {\frac{n - 0.13}{{\sqrt[3]{1 - n}}}} \right]^{2} \times (d_{10} )^{2}$	–
Chapuis	$k = 1.5 \times (d_{10} )^{2} \times \frac{{e^{3} }}{1 + e} \times \frac{{1 + e_{{\rm max} } }}{{(e_{{\rm max} } )^{3} }}$	–
Slitcher	$k = 1 \times 10^{ - 2} \times \frac{g}{v} \times n^{3.287} \times (d_{10} )^{2}$	0.01 < d_10_ < 5.0
USBR	$k = 4.8 \times 10^{ - 3} \times \frac{g}{v} \times (d_{20} )^{0.3} \times (d_{20} )^{2}$	C_u_ < 5
NAVFAC	$k = 10^{1.291e - 0.6435} \times (d_{10} )10^{{(0.5504 - 0.2937{\text{e}})}}$	2 < C_u_ < 12 0.1 < d_10_ < 2.0 0.3 < e < 0.7 $1.4 < \frac{{d_{10} }}{{d_{5} }}$
Alyamani and Sen	$k = 1300 \times [I_{0} + 0.025(d_{50} - d_{10} )]^{2}$	–
Breyer	$k = 6 \times 10^{ - 4} \times \frac{g}{v} \times \log \left[ {\frac{500}{{C_{\text{u}} }}} \right] \times (d_{10} )^{2}$	0.06 < d_10_ < 0.6 1 < C_u_ < 20

The differences between measured and predicted hydraulic conductivity values using various equations were because of either inaccuracy in measured soil parameters or deficiency in the predictive equations. Therefore, Table 5 and 6 were complied in order to present a comparative study for the NS and CSS samples using all the formulas employed in this study, respectively. The Tables 5 and 6 show the results of calculations performed with the objective of determining hydraulic conductivity according to the nine different approaches (Hazen, Slitcher, K–C, Terzaghi, USBR, Chapuis, A–S, Breyer, NAVFAC), expressed as a relative ratio of the difference between estimated and calculated values to the estimated hydraulic value of the NS and CSS samples at sixteen different gradations (4.75–2, 2–1.18, 1.18–0.6, 0.6–0.425, 0.425–0.3, 0.3–0.075, 4.75–0.075, 2–0.075, 1.18–0.075, 0.6–0.075, 0.425–0.075, 4.75–0.6, 2–0.6, 4.75–0.425, 2–0.425, 1.18–0.425 mm). The nine approaches used for comparison were listed from the best fitting on left to the worst fitting on right. For example, the best estimation of hydraulic conductivity for the NS samples between 4.75 mm and 2 mm was found to be based on Slitcher equation, followed by Terzaghi, USBR, Kozeny–Carman, Hazen, Alyamani–Sen, Chapius, Breyer, and NAVFAC equations, respectively. The authors have observed that, as an overall view, Slitcher and Terzaghi’s approaches give the best correlation with measured *k* values for both NS and CSS samples, whilst Kozeny–Carman and NAVFAC approaches give the worst correlation with measured *k* values for both NS and CSS samples for any gradation.

**Table 5:** Comparisons for the NS samples

Gradation (mm)	Approaches used for comparison from the best fitting to the worst fitting								
	1 (best)	2	3	4	5	6	7	8	9 (worst)
4.75–2	Slitcher	Terzaghi	USBR	K–C	Hazen	A–S	Chapuis	Breyer	NAVFAC
2–1.18	Slitcher	USBR	Terzaghi	Chapuis	A–S	Breyer	Hazen	K–C	NAVFAC
1.18–0.6	USBR	Slitcher	Terzaghi	Chapuis	A–S	Breyer	Hazen	K–C	NAVFAC
0.6–0.425	USBR	Slitcher	Terzaghi	Chapuis	A–S	Breyer	Hazen	K–C	NAVFAC
0.425–0.3	USBR	Slitcher	Terzaghi	Chapuis	A–S	Breyer	Hazen	K–C	NAVFAC
4.75–0.075	Slitcher	Terzaghi	NAVFAC	USBR	K–C	A–S	Chapuis	Hazen	Breyer
2–0.075	Slitcher	Terzaghi	NAVFAC	USBR	Chapuis	A–S	Breyer	K–C	Hazen
1.18–0.075	Terzaghi	Slitcher	Chapuis	A–S	NAVFAC	Breyer	USBR	Hazen	K–C
0.6–0.075	Terzaghi	Chapuis	USBR	A–S	Breyer	Slitcher	Hazen	NAVFAC	K–C
0.425–0.075	Chapuis	Breyer	Terzaghi	A–S	USBR	Slitcher	Hazen	NAVFAC	K–C
0.3–0.075	Breyer	A–S	Chapuis	Terzaghi	Hazen	Slitcher	USBR	K–C	NAVFAC
4.75–0.6	Slitcher	Terzaghi	K–C	A–S	USBR	Hazen	NAVFAC	Chapuis	Breyer
2–0.6	Slitcher	Terzaghi	USBR	Chapuis	A–S	Breyer	Hazen	K–C	NAVFAC
4.75–0.425	Slitcher	Terzaghi	K–C	NAVFAC	A–S	Hazen	Chapuis	Breyer	USBR
2–0.425	Slitcher	Terzaghi	USBR	Chapuis	A–S	Breyer	NAVFAC	Hazen	K–C
1.18–0.425	Slitcher	USBR	Terzaghi	Chapuis	A–S	Beryer	Hazen	NAVFAC	K–C

**Table 6:** Comparisons for the CSS samples

Gradation (mm)	Approaches used for comparison from the best fitting to the worst fitting								
	1 (best)	2	3	4	5	6	7	8	9 (worst)
4.75–2	Slitcher	USBR	Terzaghi	A–S	Chapuis	Breyer	Hazen	K–C	NAVFAC
2–1.18	USBR	Slitcher	Chapuis	Terzaghi	A–S	Breyer	Hazen	K–C	NAVFAC
1.18–0.6	USBR	Slitcher	Chapuis	Terzaghi	A–S	Breyer	Hazen	K–C	NAVFAC
0.6–0.425	USBR	Slitcher	Chapuis	Terzaghi	A–S	Breyer	Hazen	K–C	NAVFAC
0.425–0.3	USBR	Slitcher	Chapuis	A–S	Terzaghi	Breyer	Hazen	K–C	NAVFAC
4.75–0.075	Slitcher	Terzaghi	USBR	A–S	Chapuis	Breyer	NAVFAC	Hazen	K–C
2–0.075	Slitcher	Terzaghi	USBR	Chapuis	A–S	Breyer	Hazen	NAVFAC	K–C
1.18–0.075	Terzaghi	Slitcher	Chapuis	A–S	Breyer	USBR	Hazen	NAVFAC	K–C
0.6–0.075	Terzaghi	Chapuis	USBR	Breyer	A–S	Slitcher	Hazen	K–C	NAVFAC
0.425–0.075	Breyer	Terzaghi	Chapuis	A–S	Slitcher	Hazen	USBR	K–C	NAVFAC
0.3–0.075	Breyer	Terzaghi	Hazen	Chapuis	A–S	Slitcher	USBR	K–C	NAVFAC
4.75–0.6	Slitcher	Terzaghi	A–S	USBR	Chapuis	Breyer	Hazen	K–C	NAVFAC
2–0.6	USBR	Slitcher	Terzaghi	Chapuis	A–S	Breyer	Hazen	K–C	NAVFAC
4.75–0.425	Slitcher	Terzaghi	A–S	Chapuis	Breyer	Hazen	USBR	K–C	NAVFAC
2–0.425	Slitcher	USBR	Terzaghi	Chapuis	A–S	Breyer	Hazen	K–C	NAVFAC
1.18–0.425	USBR	Slitcher	Terzaghi	Chapuis	A–S	Breyer	Hazen	K–C	NAVFAC

**Fig. 8:**
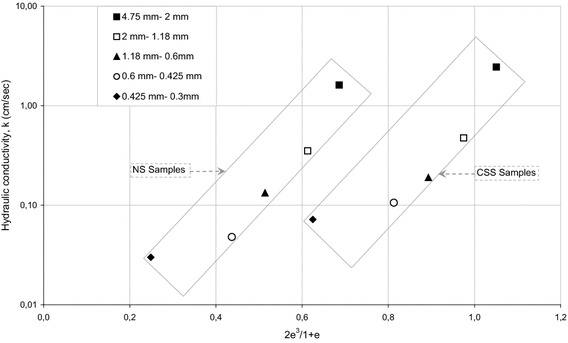
Effects of gradation on hydraulic conductivity values of NS and CSS samples

**Fig. 9:**
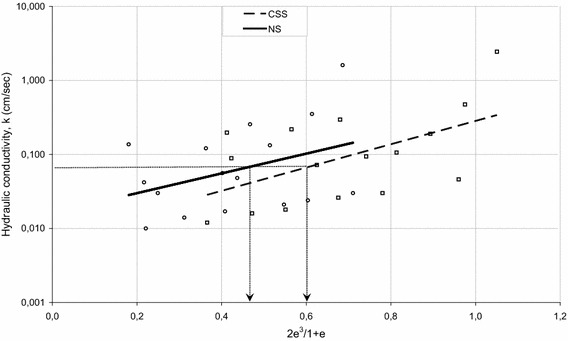
Effects of grain shape on hydraulic conductivity values of tested samples

Nevertheless, despite the good predictions in certain grading of samples, the authors interpreted that reliability of these approaches is relatively low as that any system of analysis neglecting the effect of grain shape would be incomplete. Effect of gradation as well as grain shape on hydraulic conductivity values have been presented in Figs. 8 and 9. Effects of five different gradation including 4.75–2, 2–1.18, 1.18–0.6, 0.6–0.425, and 0.425–0.3 mm on hydraulic conductivity of NS and CSS samples were illustrated in Fig. 8. The highest value of hydraulic conductivity for the NS was found to be for the samples between 4.75 and 2 mm, and then followed by the samples between 2–1.18, 1.18–0.6, 0.6–0.425, and 0.425–0.3 mm, respectively. Effects of grain shape on hydraulic conductivity values was clearly seen in Fig. 9, which proves that samples with two different shapes could have a unique hydraulic conductivity value, likely due to the differences in shape characteristics (R, S) leading to the different void ratios (e).

## Conclusions

The objective of this research was to study the influences of gradation and grain shape on hydraulic conductivity of soils, which is of importance in relation to certain geotechnical problems including stability analyses, settlement and seepage computations. The samples used in the present study are composed of poorly graded Narli Sand (NS) and Crushed Stone Sand (CSS), which were found to be rounded (R = 0.72, S = 0.67) and very angular (R = 0.15, S = 0.55), respectively. Sixteen ranges of grain sizes (4.75–2, 2–1.18, 1.18–0.6, 0.6–0.425, 0.425–0.3, 0.3–0.075, 4.75–0.075, 2–0.075, 1.18–0.075, 0.6–0.075, 0.425–0.075, 4.75–0.6, 2–0.6, 4.75–0.425, 2–0.425, and 1.18–0.425 mm) of both NS and CSS samples were tested in a constant head permeability testing apparatus at a relative density (D_r_) of about 40 %. Moreover, various predictive methods of estimating the hydraulic conductivity values (Hazen, Kozeny–Carman, Terzaghi, Chapuis, Slitcher, USBR, NAVFAC, Alyamani and Sen, and Breyer) have been employed to compare the measured and estimated hydraulic conductivity results. In general, the Slitcher and Terzaghi’s approaches give the best correlation with measured *k* values for both NS and CSS samples, whilst Kozeny–Carman and NAVFAC approaches give the worst correlation with measured *k* values for both NS and CSS samples for any gradation. The test results and comparative study reported here in this paper indicate following facets of behavior:

1. The hydraulic conductivity values of the NS samples with rounded grains were lower than those of the CSS samples with very angular grains, which is likely to be the result of shape characteristics leading different void ratios.
2. The hydraulic conductivity can be significantly influenced by grading characteristics including d_10_, d_20_, d_30_, d_50_, d_60_, c_u_, c_c_, n, and I_o_.
3. Gradation of the grains have a significant effect on hydraulic conductivity of both NS and CSS samples.
4. The comparative study on the perceptions of estimated and predicted results with other approaches available in the literature indicated that the best prediction of hydraulic conductivity changes based on the gradation and shape properties of the sands tested.
